# Real-world clinical outcomes and safety of anlotinib-containing regimens in pediatric solid tumors: a retrospective cohort study at a Chinese center

**DOI:** 10.3389/fonc.2026.1722123

**Published:** 2026-09-25

**Authors:** Qiaoli Hua, Zhouqi Xia, Yaqing Gu, Jinhu Wang, Shaoqing Ni

**Affiliations:** 1 Research Center for Clinical Pharmacy, College of Pharmaceutical Sciences, Zhejiang University, Hangzhou, Zhejiang, China; 2 National Clinical Trial Institute, Children’s Hospital Zhejiang University School of Medicine, National Clinical Research Center for Child Health, Hangzhou, Zhejiang, China; 3 Pediatric Cancer Research Center, National Clinical Research Center for Child Health, Hangzhou, Zhejiang, China; 4 Department of Surgical Oncology, Children’s Hospital Zhejiang University School of Medicine, National Clinical Research Center for Child Health, Hangzhou, Zhejiang, China; 5 Key Laboratory of Diagnosis and Treatment of Neonatal Diseases of Zhejiang Province, Hangzhou, Zhejiang, China; 6 Cancer Center, Zhejiang University, Hangzhou, Zhejiang, China

**Keywords:** anlotinib, child, cohort studies, neoplasms, patient safety, sarcoma, treatment outcome

## Abstract

**Background and aim:**

This retrospective cohort study aimed to characterize the real-world clinical outcomes and treatment-emergent adverse events (TEAEs) of anlotinib-containing regimens among pediatric patients with solid tumors treated at the Children’s Hospital of Zhejiang University School of Medicine from January 2020 to May 2025.

**Methods:**

This retrospective cohort study included 45 eligible patients treated with anlotinib at the Children’s Hospital of Zhejiang University School of Medicine from January 2020 to May 2025; eligibility required age <18 years at solid tumor diagnosis, first-time anlotinib use at our center, RECIST 1.1-measurable disease, and available clinical and imaging data. Progression-free survival (PFS) was the primary endpoint, and objective response rate (ORR), disease control rate (DCR), and overall survival (OS) were secondary endpoints. Safety was assessed using the Common Terminology Criteria for Adverse Events version 5.0 (CTCAE v5.0).

**Results:**

The observed ORR was 8.9% (4/45), and the DCR was 40.0% (18/45; 4 partial responses and 14 cases of stable disease). The median PFS was 5.6 months (95% CI: 2.9–8.3), while the median OS was not reached after seven deaths. Patients with other solid tumors (n=21) had an ORR of 19.0%, a DCR of 57.1%, and a median PFS of 9.4 months, compared with 0%, 25.0%, and 3.0 months, respectively, among patients with sarcomas (n=24). The observed DCR was 80.0% among patients without metastatic or recurrent disease (n=10) and 28.6% among those with metastatic or recurrent disease (n=35). DCRs were 33.3%, 48.0%, and 0% in the usual-dose (n=18), low-dose (n=25), and high-dose (n=2) groups, respectively; exploratory analyses did not detect statistically significant differences in DCR (p=0.393) or PFS distributions (p=0.395) across the three groups. Exploratory analyses did not detect statistically significant differences across the treatment-regimen groups in ORR (p=0.133), DCR (p=0.454), or PFS distributions (p=0.480). TEAEs were documented in 97.8% of patients, with grade 3–4 TEAEs observed in 84.4%. Hematologic TEAEs were the most frequently documented category (91.1%). Frequent non-hematologic events included pyrexia (62.2%), proteinuria (35.6%), hypokalemia (33.3%), and hypoalbuminemia (33.3%).

**Conclusion:**

In this small, heterogeneous, single-center Chinese pediatric cohort, patients receiving anlotinib-containing regimens had limited objective response and a high incidence of grade 3–4 TEAEs, predominantly involving hematologic events. Subgroup findings were exploratory and may reflect selection bias, confounding by indication, tumor heterogeneity, and concomitant therapies. Larger prospective studies are required before comparative treatment or dosing conclusions can be made.

## Introduction

Pediatric cancer represents a substantial global burden, with an estimated 400,000 children and adolescents aged 0–19 years developing cancer annually (1). In China, a national study estimated 121,145 incident cancers among children aged 0–14 years and adolescents aged 15–19 years during 2018–2020, with cases distributed across 12 main diagnostic groups (2). Pediatric solid tumors encompass a heterogeneous spectrum of central nervous system tumors and extracranial malignancies, including neuroblastoma, retinoblastoma, renal and hepatic tumors, bone and soft-tissue sarcomas, and germ cell tumors (2). Despite advances in multimodal therapy, relapsed or refractory pediatric solid tumors continue to have poor outcomes and limited treatment options (3), underscoring the need for new treatment strategies. At our tertiary pediatric oncology center, anlotinib is commonly considered in real-world salvage settings for children with relapsed, refractory, metastatic, or heavily pretreated solid tumors after standard therapies.

Anlotinib is an orally administered multitargeted tyrosine kinase inhibitor developed in China. It inhibits vascular endothelial growth factor receptors 1–3, fibroblast growth factor receptors 1–3, platelet-derived growth factor receptor-α, and c-Kit, thereby targeting tumor angiogenesis and proliferation (4). In May 2018, the China National Medical Products Administration approved anlotinib for patients with advanced non-small cell lung cancer who had progressed after at least two prior lines of systemic chemotherapy; the adult regimen used in pivotal development was 12 mg once daily on days 1–14 of a 21-day cycle (5). Clinical activity has also been investigated in adults with refractory metastatic soft tissue sarcoma (6) and locally advanced or metastatic medullary thyroid carcinoma (7).

Anlotinib use in pediatric solid tumors remains off-label, and the available clinical evidence is limited. In a single-center retrospective cohort of refractory or recurrent pediatric solid tumors, the objective response rate (ORR) was 12.2%, the disease control rate (DCR) was 65.9%, the median progression-free survival (PFS) was 2.87 months, and the incidence of grade 3–4 adverse events (AEs) was 23.3% (3). A subsequent single-center phase Ia/Ib trial in patients aged 5–18 years with high-risk, recurrent, or refractory sarcomas identified a maximum tolerated dose and recommended phase II dose of 8 mg for patients weighing <35 kg and 12 mg for those weighing ≥35 kg; in the 13-patient maintenance cohort, the 2-year PFS was 84.6% (8). This maintenance population and its landmark 2-year PFS estimate are not directly comparable with median PFS reported in salvage-treatment cohorts.

Although these studies provide important preliminary data, both were small, single-center studies and enrolled clinically distinct populations (3, 8). Their findings cannot establish comparative effectiveness across tumor types or a universal pediatric dose. Larger multicenter prospective studies are needed to characterize dose-exposure relationships, long-term safety, and clinical outcomes across pediatric solid tumors.

However, real-world evidence on anlotinib in Chinese pediatric patients with solid tumors remains limited, particularly regarding heterogeneous tumor types, combination regimens, dose patterns, and treatment-emergent adverse events. Therefore, we conducted this retrospective cohort study to describe the real-world clinical outcomes and treatment-emergent adverse events (TEAEs) among pediatric patients with solid tumors who received anlotinib-containing regimens at our center from January 2020 to May 2025.

## Patients and methods

### Study design and setting

This was a single-center retrospective cohort study of pediatric patients with solid tumors who received anlotinib at the Children’s Hospital of Zhejiang University School of Medicine (ZCH) from January 2020 to May 2025. Because the study included all available patients who met the prespecified eligibility criteria during the study period, no formal sample size calculation was performed; the final cohort represented a census sample of all eligible cases at our center. The study was reported in accordance with the Strengthening the Reporting of Observational Studies in Epidemiology (STROBE) statement for cohort studies (9).

### Eligibility criteria

The inclusion criteria were as follows: (1) patients aged under 18 years at the time of solid tumor diagnosis; (2) first-time anlotinib administration at ZCH; (3) presence of at least one measurable lesion, assessable via computed tomography (CT) or magnetic resonance imaging (MRI) according to the Response Evaluation Criteria in Solid Tumors, version 1.1 (RECIST 1.1) (10); and (4) availability of baseline clinical information, at least one post-treatment imaging assessment suitable for RECIST 1.1 evaluation, and outcome and safety records sufficient for the prespecified analyses. The exclusion criteria were: (1) receipt of fewer than one complete 21-day cycle of anlotinib-containing treatment; or (2) absence of a post-treatment imaging assessment or follow-up information sufficient to evaluate treatment response and derive valid survival outcomes. Eligibility age was defined at solid tumor diagnosis, whereas baseline age was summarized at anlotinib initiation.

### Study variables and definitions

The pediatric population was operationally defined as patients aged <18 years at solid tumor diagnosis, consistent with the eligibility criterion described above. Solid tumors were defined as malignant non-hematologic neoplasms involving the central nervous system (CNS) or extracranial sites; leukemias and lymphomas were excluded, consistent with the diagnostic grouping used in the national Chinese pediatric cancer study (2). For subgroup analyses, metastatic or recurrent disease was defined as documented metastatic disease and/or documented tumor recurrence present at anlotinib initiation. Patients with neither condition were classified as patients without metastatic or recurrent disease. Refractory status was not consistently available as a separately coded variable in the retrospective records and was therefore not included in this classification. The available dataset did not permit reliable reconstruction of mutually exclusive metastatic, recurrent, and refractory subgroups; these conditions were therefore not analyzed separately. Radiologically measurable disease was required according to RECIST 1.1 (10). For the histology subgroup analyses, patients with rhabdomyosarcoma, alveolar soft part sarcoma, Ewing sarcoma, desmoplastic small round cell tumor, or undifferentiated small round cell sarcoma were categorized as patients with sarcomas; all remaining histologies were categorized as other solid tumors. For grouping analyses, “unmodified regimen” referred to the initial anlotinib-containing regimen recorded at treatment initiation, before any subsequent dose adjustment, interruption, or discontinuation. The regimen categories were anlotinib monotherapy, anlotinib plus immune checkpoint inhibitors, anlotinib plus chemotherapy, anlotinib plus chemo-immunotherapy, and anlotinib plus targeted therapy.

### Data collection and ethics

Data were retrospectively extracted from hospital electronic medical records according to the prespecified study variables. The extracted data included demographics, tumor diagnosis and histology, prior anticancer treatment, anlotinib-containing regimen, actual anlotinib dose, imaging-based treatment response, survival outcomes, follow-up status, and adverse event (AE) type, severity, and management. The extracted dataset was checked against the source medical records for completeness and consistency before statistical analysis. No statistical imputation was performed. Among the 45 patients included in the final analytic cohort, no values were missing for age at anlotinib initiation, sex, histologic classification, metastatic or recurrent disease status, initial treatment regimen, initial dose category, response assessment, PFS, OS, or TEAE status. Variables that were not systematically captured in the retrospective electronic medical records were not reconstructed and were excluded from the corresponding analyses. Follow-up duration was calculated from the date of initial anlotinib treatment to the date of last documented clinical follow-up or death. The data cutoff for the survival analyses was May 16, 2025. The study was conducted in accordance with the Declaration of Helsinki and was approved by the Ethics Committee of the Children’s Hospital of Zhejiang University School of Medicine (No. 2024-IRB-0022). The requirement for written informed consent from patients or their legal guardians was waived by the ethics committee because only retrospective anonymized data were used.

### Treatment schedule

Anlotinib was administered orally once daily for 14 consecutive days followed by a 7-day break (21-day cycle). Because pediatric anlotinib dosing remains investigational and the available evidence is limited to small studies (3, 8), initial treatment decisions and doses were determined through multi-physician review based on clinical status, prior therapy, and anticipated tolerance. Treatment regimens encompassed five categories: anlotinib monotherapy (Group A), anlotinib combined with an immune checkpoint inhibitor (ICI) (Group B), anlotinib plus chemotherapy (Group C), anlotinib with both chemotherapy and immunotherapy (Group D), and anlotinib plus targeted therapy (Group E). For analysis, patients were grouped according to the initial anlotinib-containing regimen recorded at anlotinib initiation. The ICI agents used were four programmed cell death protein 1 (PD-1) inhibitors: sintilimab, penpulimab, tislelizumab, and camrelizumab. Chemotherapy regimens included irinotecan plus carboplatin, vindesine combined with cyclophosphamide and epirubicin, and cisplatin with gemcitabine, among others. Targeted therapies included bevacizumab and lorlatinib. For dose-group analyses, patients were categorized according to their initial anlotinib dose relative to the locally used weight-banded regimen: usual dose (UD, operationally defined for this study as 8 mg for patients weighing <35 kg or 10 mg for those weighing ≥35 kg), low dose (LD, below UD), and high dose (HD, above UD). This operational definition reflected local prescribing practice during the study period and was consistent with the pediatric dosing schedule previously reported by Lu et al. (3). The subsequent phase Ia/Ib dose-finding trial identified a recommended phase II dose of 8 mg for patients weighing <35 kg and 12 mg for those weighing ≥35 kg (8). In routine clinical practice at our center, anlotinib was administered using a weight-banded fixed-dose approach, with the prescribed dose determined by the patient’s body-weight category rather than calculated in mg/kg or mg/m².

### Assessment of response and toxicity

For this single-center retrospective cohort, we described clinical outcomes and TEAEs among pediatric patients with solid tumors who received anlotinib-containing regimens at ZCH from January 2020 to May 2025. PFS was the primary endpoint; ORR, DCR, and OS were secondary endpoints. Response and progression assessments were based on routine CT and MRI reports retrieved from the hospital electronic medical record system. These reports were independently reviewed by two pediatric surgical oncologists and one radiologist at ZCH. The reviewers were blinded to the patients’ treatment regimens and subsequent clinical outcomes. Any disagreement in response or progression classification was resolved through joint discussion until consensus was reached. A *de novo* reassessment of the original images was not performed. We also explored factors potentially associated with treatment response, including histologic subtype, treatment regimen, and anlotinib dose.

Tumor response was evaluated according to RECIST 1.1 and classified as complete response (CR), partial response (PR), stable disease (SD), or progressive disease (PD) (10). CR was defined as the disappearance of all target lesions and a reduction in the short axes of all pathological lymph nodes to <10 mm. PR was defined as a decrease of at least 30% in the sum of target-lesion diameters relative to baseline. PD was defined as an increase of at least 20% in the sum of target-lesion diameters relative to the smallest sum recorded during the study, with an absolute increase of at least 5 mm, or the appearance of one or more new lesions. SD was defined as a response meeting neither the criteria for PR nor those for PD.

RECIST 1.1 was applied uniformly to maintain a consistent response-assessment framework across this histologically heterogeneous retrospective cohort. Because the standardized imaging sequences and accompanying clinical variables required to retrospectively apply disease-specific Response Assessment in Neuro-Oncology (RANO) or Response Assessment in Pediatric Neuro-Oncology (RAPNO) criteria were not uniformly available, these criteria could not be applied consistently across the CNS subgroup (11).

ORR was defined as the proportion of patients achieving CR or PR, whereas DCR included CR, PR, and SD. PFS was defined as the time from initial anlotinib treatment to disease progression or death, and OS as the time from initial anlotinib treatment to death from any cause. For PFS, patients without documented progression or death were censored on the date of their last documented follow-up without progression; death without prior documented radiological progression was counted as a PFS event on the date of death. For OS, patients who were alive were censored on the date they were last known to be alive.

Safety was described using treatment-emergent adverse events (TEAEs), which were graded according to the Common Terminology Criteria for Adverse Events version 5.0 (CTCAE v5.0) (12) and coded using the Medical Dictionary for Regulatory Activities (MedDRA) (13) by System Organ Class (SOC) and Preferred Term (PT). TEAEs were defined as adverse events documented from the first dose through 34 days after the last dose, regardless of their causal relationship with anlotinib. The 34-day observation window was derived conservatively as approximately five times the upper bound of the reported terminal elimination half-life estimate (116 ± 47 hours): 5 × (116 + 47) hours ≈ 34 days (14). Events occurring more than 34 days after the last dose fell outside the prespecified TEAE observation window and were therefore not included; no separate treatment-relatedness determination was applied to such events.

TEAE incidence was calculated according to the number of affected patients. At the PT level, each patient was counted once for each PT according to the highest CTCAE grade recorded during the observation window. At the SOC level, the any-grade count represented the number of unique patients with at least one TEAE within that SOC. A patient could contribute to both the Grade 1–2 and Grade 3–4 columns when different PTs within the same SOC had different maximum grades. Consequently, the two severity columns at the SOC level could sum to more than the any-grade count. Continuous monitoring of blood pressure and hematologic parameters was necessary throughout treatment.

### Statistical methods

Categorical variables were summarized as counts and percentages, and continuous variables were summarized as medians and ranges. Two-sided 95% confidence intervals for ORR and DCR were calculated using the exact Clopper–Pearson method. For subgroup comparisons, ORR and DCR were analyzed as binary outcomes. Two-sided Fisher’s exact test was used for 2 × 2 tables, and the two-sided Fisher–Freeman–Halton exact test was used for tables containing more than two groups. Survival curves and median survival times with corresponding 95% CIs were estimated using the Kaplan–Meier method, and survival distributions were compared using two-sided log-rank tests. A *post hoc* sensitivity analysis of ORR, DCR, and PFS was performed after excluding patients with primary CNS tumors. Given the sample size (n=45), the occurrence of only four objective responses, the marked subgroup imbalance, and the number of clinically relevant potential confounders, no multivariable Cox or logistic regression model was fitted because reliable adjusted estimates could not be obtained. All subgroup analyses were therefore unadjusted and exploratory. No adjustment for multiplicity was applied; subgroup p values were interpreted descriptively rather than as confirmatory evidence. Statistical analyses were performed using IBM SPSS Statistics, version 27.0 (IBM Corp., Armonk, NY, USA). All p values were two-sided; p < 0.05 was considered nominally significant, and subgroup findings were interpreted as exploratory.

## Results

### Patient characteristics

Between January 2020 and May 2025, a total of 52 potentially eligible patients were screened through the hospital electronic medical records. After applying the prespecified eligibility criteria, 45 patients were included in the final evaluable cohort. Seven patients were excluded: five lacked post-treatment imaging required for RECIST 1.1-based response assessment, and two received fewer than one cycle of anlotinib treatment. For these seven patients, the available records did not contain documented progression or death dates or adequate post-baseline disease assessments from which valid PFS event or censoring times could be determined; therefore, they were excluded before formation of the final analytic cohort and were not included in the response, survival, or safety analyses. The participant screening and analysis process is shown in **Figure 1**. Baseline demographic and clinical characteristics are summarized in **Table 1**. Available baseline and initial treatment characteristics stratified by the initial anlotinib-containing regimen are provided in **Supplementary Table S1**. The cohort had a median age of 7.2 years (range: 1.1–19.0) at initial anlotinib treatment, and 26 patients were male (57.8%). Histopathological subtypes included rhabdomyosarcoma (n=18), nephroblastoma (n=6), diffuse glioma (n=6), neuroblastoma (n=5), alveolar soft part sarcoma (n=2), and Ewing sarcoma (n=2), along with single cases of ependymoma, desmoplastic small round cell tumor, medulloblastoma, sex cord-stromal tumor, hepatoblastoma, and undifferentiated small round cell sarcoma. Metastatic or recurrent disease was present in 35 patients (77.8%) at the time of initial anlotinib treatment. The median follow-up duration was 7.1 months (range: 0.5–52.8 months). All patients had received prior anticancer therapies, including surgery (n=17, 37.8%), radiotherapy (n=14, 31.1%), chemotherapy, or combination regimens.

**Figure 1:**
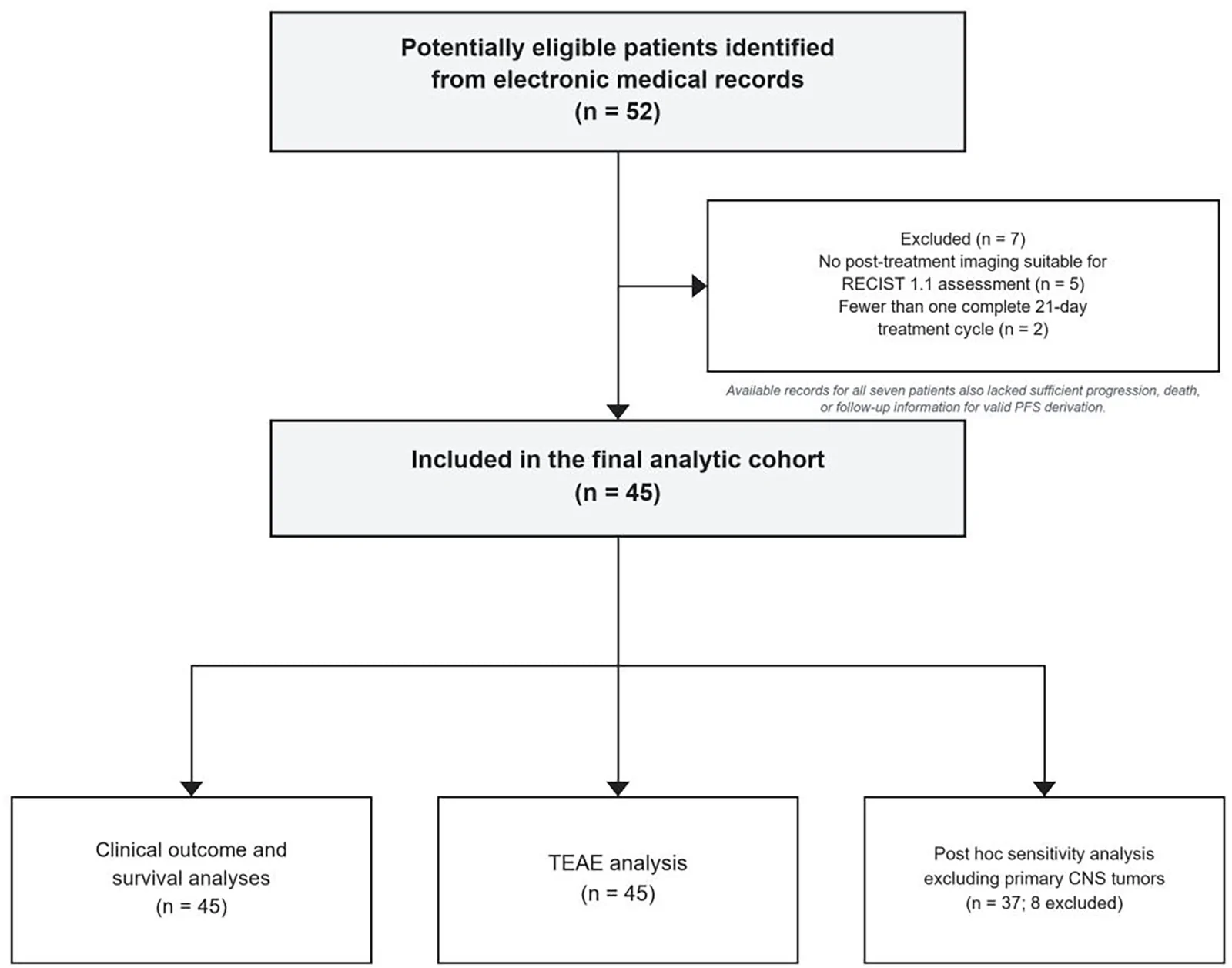
Participant flow diagram. CNS, central nervous system; RECIST 1.1, Response Evaluation Criteria in Solid Tumors version 1.1; TEAE, treatment-emergent adverse event.

**Table 1:** Baseline demographic and clinical characteristics of pediatric patients with solid tumors receiving anlotinib (n=45).

Characteristics	Total (n=45), n (%)
Age at anlotinib initiation, median (range), years	7.2 (1.1–19.0)
Sex	
Male	26 (57.8%)
Female	19 (42.2%)
Histology	
Rhabdomyosarcoma	18 (40%)
Nephroblastoma	6 (13.3%)
Diffuse glioma	6 (13.3%)
Neuroblastoma	5 (11.1%)
Alveolar soft part sarcoma	2 (4.4%)
Ewing sarcoma	2 (4.4%)
Ependymoma	1 (2.2%)
Desmoplastic small round cell tumor	1 (2.2%)
Medulloblastoma	1 (2.2%)
Sex cord-stromal tumor	1 (2.2%)
Hepatoblastoma	1 (2.2%)
Undifferentiated small round cell sarcoma	1 (2.2%)
Metastatic or recurrent disease at anlotinib initiation	
Yes	35 (77.8%)
No	10 (22.2%)
Follow-up duration, median (range), months	7.1 (0.5–52.8)

Age was recorded at initial anlotinib treatment, whereas eligibility was based on age at solid tumor diagnosis. The patient aged 19.0 years at anlotinib initiation had been diagnosed with rhabdomyosarcoma at 16 years of age.

### Clinical and survival outcomes

Following at least one treatment cycle of anlotinib-based therapy in 45 evaluable patients, no complete responses were observed. Partial response occurred in 4 patients (8.9%), stable disease in 14 (31.1%), and progressive disease in 27 (60%), yielding an ORR of 8.9% (95% confidence interval [CI]: 2.5–21.2) and a DCR of 40% (95% CI: 25.7–55.7). The median PFS was 5.6 months (95% CI: 2.9–8.3). At the data cutoff of May 16, 2025, 28 PFS events had occurred (27 radiologically documented progressions and one death without prior documented radiological progression), and 17 patients (37.8%) were censored for PFS. Seven deaths had occurred; 38 patients (84.4%) were censored for OS, and the median OS and its 95% CI were not estimable. The dynamics of treatment responses and the timelines of clinical events across the cohort are illustrated in **Figure 2**.

**Figure 2:**
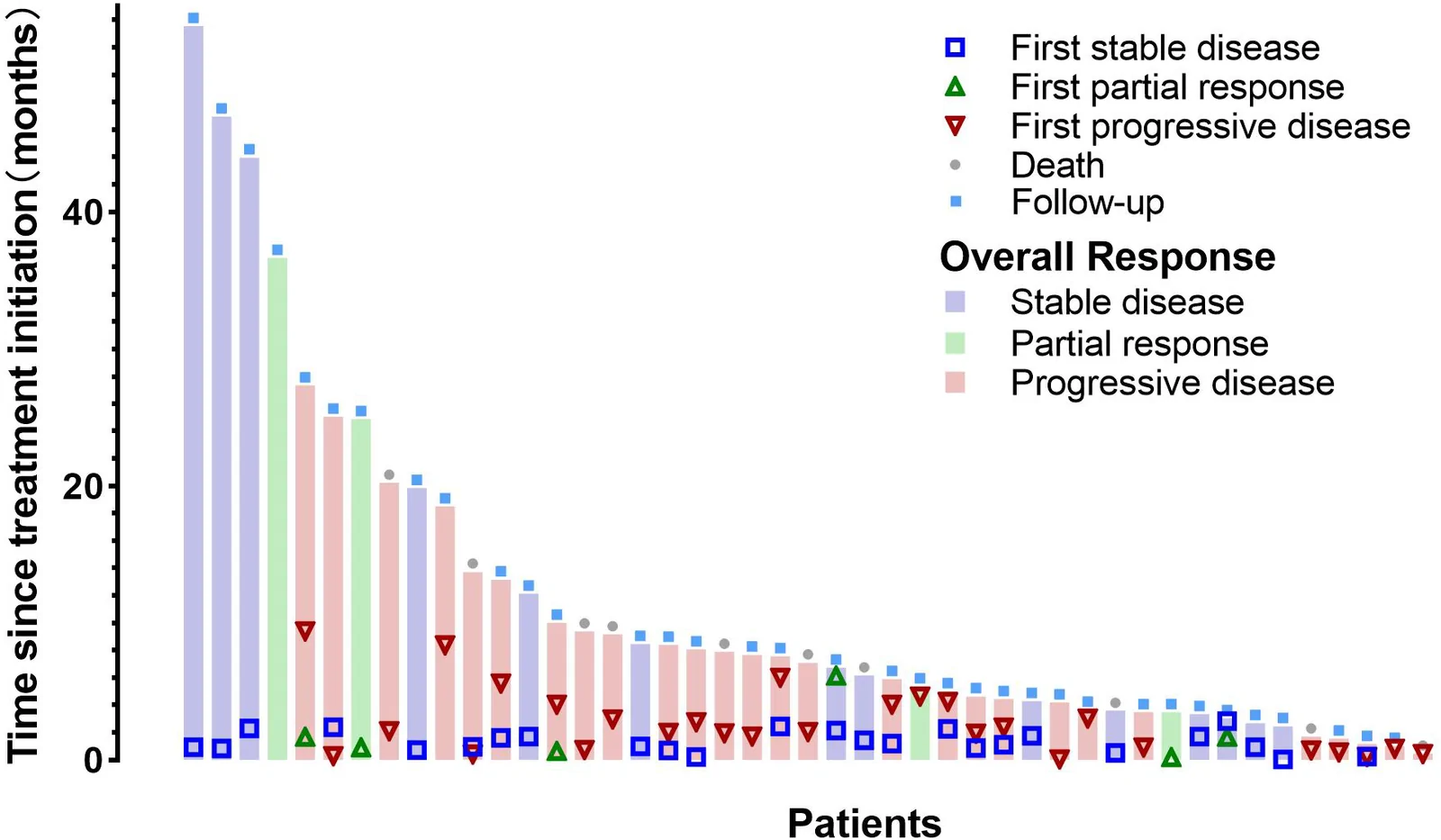
Swimlane plot of longitudinal treatment response, disease progression, and survival events in pediatric patients with solid tumors treated with anlotinib (RECIST 1.1; independent review).

Eight patients had primary CNS tumors, including six with diffuse glioma, one with ependymoma, and one with medulloblastoma. In a *post hoc* sensitivity analysis excluding these patients, the remaining 37 patients had an ORR of 5.4% (2/37; 95% CI: 0.7–18.2), a DCR of 29.7% (11/37; 95% CI: 15.9–47.0), and a median PFS of 4.1 months. These estimates were numerically lower than those of the complete cohort and remained consistent with the overall interpretation of limited objective response.

When stratified by histology, patients with sarcomas (n=24) had an ORR of 0% (95% CI: 0–14.2), a DCR of 25.0% (95% CI: 9.8–46.7), and a median PFS of 3.0 months, whereas patients with other solid tumors (n=21) had an ORR of 19.0% (95% CI: 5.4–41.9), a DCR of 57.1% (95% CI: 34.0–78.2), and a median PFS of 9.4 months. Exploratory subgroup comparisons yielded nominal p values of 0.040 for ORR, 0.037 for DCR, 0.073 for PFS distributions, and 0.181 for OS distributions (**Figures 3A, B**).

**Figure 3:**
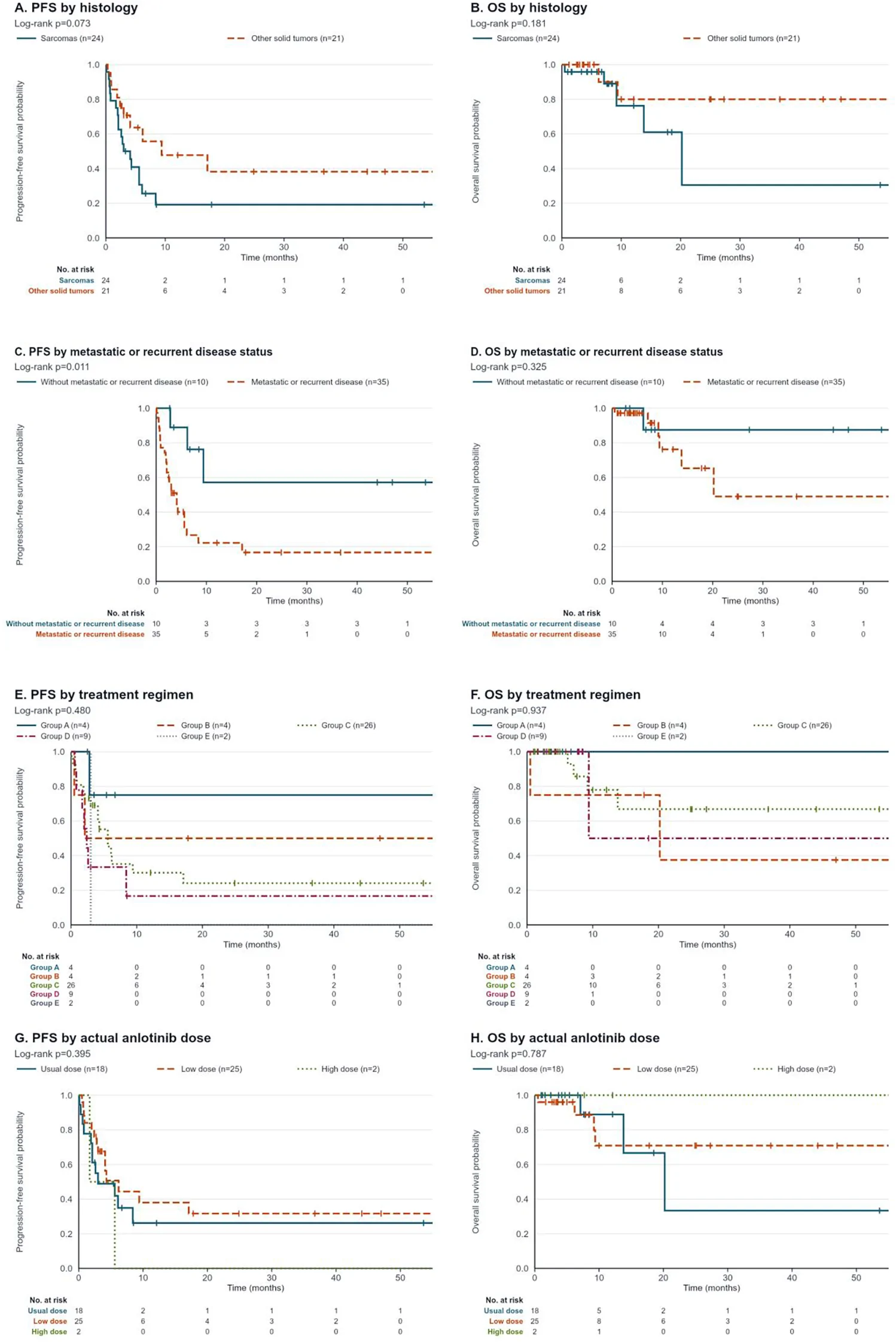
Kaplan–Meier analysis of progression-free survival (PFS) and overall survival (OS) stratified by clinical subgroups; **(A)** PFS by histology, **(B)** OS by histology, **(C)** PFS by metastatic or recurrent disease status, **(D)** OS by metastatic or recurrent disease status, **(E)** PFS by treatment regimen, **(F)** OS by treatment regimen, **(G)** PFS by actual anlotinib dose, and **(H)** OS by actual anlotinib dose. Due to the extremely small sample sizes in certain subgroups (such as Groups A, B, and E), their corresponding survival curves are provided strictly for descriptive visualization. Statistical estimates and log-rank p values for these specific groups are highly unstable and should not be interpreted as definitive comparative data. Numbers at risk are shown below each curve at 0, 10, 20, 30, 40, and 50 months. Crosses indicate censored observations, and displayed p values were derived from two-sided log-rank tests.

Patients with metastatic or recurrent disease (n=35) demonstrated 8.6% ORR (95% CI 1.8–23.1), 28.6% DCR (95% CI 14.6–46.3), and median PFS of 4.1 months. Patients without metastatic or recurrent disease (n=10) achieved 10% ORR (95% CI 0.3–44.5), 80% DCR (95% CI 44.4–97.5), and median PFS not reached. Exploratory subgroup comparisons yielded nominal p values of 1.000 for ORR, 0.008 for DCR, 0.011 for PFS distributions, and 0.325 for OS distributions (**Figures 3C, D**).

Treatment responses by regimen group are comprehensively summarized in **Table 2**. Median PFS was not reached in Group A, while Groups B, C, D, and E demonstrated median PFS of 2.1, 5.6, 2.4, and 3.0 months, respectively. Exploratory comparisons across the treatment groups yielded nominal p values of 0.133 for ORR, 0.454 for DCR, 0.480 for PFS distributions, and 0.937 for OS distributions (**Figures 3E, F**).

**Table 2:** Treatment responses according to the initial anlotinib-containing regimen: monotherapy (Group A), ICI combination (Group B), chemotherapy combination (Group C), chemotherapy plus immunotherapy (Group D), and targeted-therapy combination (Group E).

Clinical evaluations	Overall patients	Group A	Group B	Group C	Group D	Group E
Total, n	45	4	4	26	9	2
CR, n	0	0	0	0	0	0
PR, n (%)	4 (8.9%)	2 (50%)	0	2 (7.7%)	0	0
SD, n (%)	14 (31.1%)	1 (25%)	2 (50%)	8 (30.8%)	2 (22.2%)	1 (50%)
PD, n (%)	27 (60%)	1 (25%)	2 (50%)	16 (61.5%)	7 (77.8%)	1 (50%)
ORR (%, 95% CI)	8.9% (2.5–21.2)	50% (6.8–93.2)	0 (0–60.2)	7.7% (0.9–25.1)	0 (0–33.6)	0 (0–84.2)
DCR (%, 95% CI)	40% (25.7–55.7)	75% (19.4–99.4)	50% (6.8–93.2)	38.5% (20.2–59.4)	22.2% (2.8–60.0)	50% (1.3–98.7)

CR, complete response; PR, partial response; SD, stable disease; PD, progressive disease; ORR, objective response rate; DCR, disease control rate; CI, confidence interval. Two-sided 95% CIs for ORR and DCR were calculated using the exact Clopper–Pearson method.

Because of the small, markedly unbalanced regimen groups and substantial clinical heterogeneity, these unadjusted results are presented strictly as descriptive observations and should not be interpreted as comparative effectiveness estimates.

Stratified by actual anlotinib dose, the UD, LD, and HD cohorts achieved ORRs of 5.6% (95% CI 0.1–27.3), 12% (95% CI 2.5–31.2), and 0% (95% CI 0–84.2), respectively (p=0.692). The corresponding DCRs were 33.3% (95% CI 13.3–59.0), 48% (95% CI 27.8–68.7), and 0% (95% CI 0–84.2), respectively (p=0.393). Median PFS was 3.0, 6.2, and 1.7 months in the UD, LD, and HD cohorts, respectively, with nominal p values of 0.395 for PFS distributions and 0.787 for OS distributions (**Figures 3G, H**).

### Safety and treatment-emergent adverse events

Safety and toxicity were evaluated in all 45 patients included in the final analytic cohort, each of whom received at least one dose of anlotinib and had evaluable safety documentation. TEAEs occurred in 44 patients (97.8%), with grade 3–4 events observed in 38 patients (84.4%). SOCs affecting >50% of patients included blood and lymphatic system disorders (91.1%), metabolism and nutrition disorders (82.2%), general disorders and administration site conditions (64.4%), and gastrointestinal disorders (51.1%). Incidence rates (≥20%) are presented by SOC and PT in **Table 3**. Detailed safety data for all specific treatment subgroups (Groups A–E) are provided in **Supplementary Table S2**. Hematologic TEAEs were documented in 41 patients (91.1%), with anemia (88.9%), leukopenia (84.4%), and thrombocytopenia (68.9%) being the most frequent. Among these 41 patients, 35 (85.4%) experienced at least one grade 3–4 hematologic TEAE. Other common TEAEs included pyrexia (62.2%), proteinuria (35.6%), hypokalemia (33.3%), hypoalbuminemia (33.3%), and hyponatremia (33.3%). Dose reduction or discontinuation of anlotinib was documented in three patients in association with grade 3 hypertension, proteinuria, and palmar-plantar erythrodysesthesia with arthralgia; these events resolved following clinical management. The available medical records did not identify any death as AE-related. Because this was a retrospective electronic medical record-based study, baseline laboratory abnormalities, treatment interruptions or delays, hospitalizations, transfusions, growth-factor support, and serious adverse events were not consistently retrievable for reliable cohort-level reporting.

**Table 3:** Incidence and severity of treatment-emergent adverse events (TEAEs) occurring in ≥20% of pediatric patients with solid tumors receiving anlotinib-containing regimens, categorized by system organ class and preferred term.

TEAEs	Any grade	Grade 1–2	Grade 3–4
**Blood and lymphatic system disorders**	**41 (91.1%)**	**24 (53.3%)**	**35 (77.8%)**
Anemia	40 (88.9%)	12 (26.7%)	28 (62.2%)
White blood cell decreased	38 (84.4%)	9 (20%)	29 (64.4%)
Platelet count decreased	31 (68.9%)	3 (6.7%)	28 (62.2%)
Neutrophil count decreased	30 (66.7%)	4 (8.9%)	26 (57.8%)
Activated partial thromboplastin time prolonged	11 (24.4%)	8 (17.8%)	3 (6.7%)
Lymphocyte count decreased	10 (22.2%)	1 (2.2%)	9 (20%)
**Metabolism and nutrition disorders**	**37 (82.2%)**	**35 (77.8%)**	**10 (22.2%)**
Hypokalemia	15 (33.3%)	12 (26.7%)	3 (6.7%)
Hypoalbuminemia	15 (33.3%)	13 (28.9%)	2 (4.4%)
Hyponatremia	15 (33.3%)	14 (31.1%)	1 (2.2%)
Hypertriglyceridemia	13 (28.9%)	11 (24.4%)	2 (4.4%)
Hypocalcemia	12 (26.7%)	9 (20%)	3 (6.7%)
Blood lactate dehydrogenase increased	11 (24.4%)	8 (17.8%)	3 (6.7%)
Hypophosphatemia	11 (24.4%)	11 (24.4%)	0
Hyperglycemia	11 (24.4%)	11 (24.4%)	0
Hypomagnesemia	10 (22.2%)	10 (22.2%)	0
**General disorders and administration site conditions**	**29 (64.4%)**	**26 (57.8%)**	**4 (8.9%)**
Pyrexia	28 (62.2%)	25 (55.6%)	3 (6.7%)
Gastrointestinal disorders	**23 (51.1%)**	**20 (44.4%)**	**6 (13.3%)**
Vomiting	12 (26.7%)	9 (20%)	3 (6.7%)
Nausea	9 (20%)	8 (17.8%)	1 (2.2%)
Gastrointestinal hemorrhage	9 (20%)	9 (20%)	0
**Hepatobiliary disorders**	**20 (44.4%)**	**18 (40%)**	**3 (6.7%)**
Gamma-glutamyltransferase increased	13 (28.9%)	12 (26.7%)	1 (2.2%)
Aspartate aminotransferase increased	9 (20%)	7 (15.6%)	2 (4.4%)
**Renal and urinary disorders**	**17 (37.8%)**	**15 (33.3%)**	**5 (11.1%)**
Proteinuria	16 (35.6%)	13 (28.9%)	3 (6.7%)
Hematuria	12 (26.7%)	8 (17.8%)	4 (8.9%)
**Cardiac disorders**	**20 (44.4%)**	**20 (44.4%)**	**0**
Sinus tachycardia	14 (31.1%)	14 (31.1%)	0

TEAE, treatment-emergent adverse event; SOC, System Organ Class; PT, Preferred Term. At the PT level, each patient was counted once per PT according to the highest recorded CTCAE grade; therefore, the Grade 1–2 and Grade 3–4 counts sum to the any-grade count. At the SOC level, the any-grade value represents unique patients with at least one TEAE in that SOC. A patient could contribute to both severity columns when different PTs within the same SOC had different maximum grades; therefore, the Grade 1–2 and Grade 3–4 counts may sum to more than the any-grade SOC count. TEAEs with an overall incidence below 20% are not displayed in this table. Bold values indicate System Organ Class (SOC)-level counts; non-bold values indicate Preferred Term (PT)-level counts.

Detailed TEAE frequencies according to the initial anlotinib-containing regimen are provided in **Supplementary Table S2**. Grade 3–4 hematologic TEAEs were documented in 2/4 patients in Group A, 3/4 in Group B, 19/26 in Group C, 9/9 in Group D, and 2/2 in Group E. Grade 3–4 non-hematologic TEAEs were documented in 2/4, 2/4, 10/26, 6/9, and 2/2 patients, respectively. Given the small and markedly unbalanced groups, particularly Groups A, B, and E, and the differences in concomitant therapies, these frequencies are presented as descriptive observations only and do not support causal, comparative, or mechanistic interpretation.

## Discussion

### Clinical outcomes and disease context

This real-world study described clinical outcomes among 45 pediatric patients with solid tumors who received anlotinib-containing regimens. The observed ORR was 8.9%, the DCR was 40%, and the median PFS was 5.6 months; OS data remained immature, with seven deaths observed and the median OS not reached. These outcomes should be interpreted in the context of the tertiary referral setting: 77.8% of patients had metastatic or recurrent disease, and all had received prior anticancer therapy. Patients with sarcomas (n=24) had an ORR of 0%, a DCR of 25.0%, and a median PFS of 3.0 months, whereas patients with other solid tumors (n=21) had an ORR of 19.0%, a DCR of 57.1%, and a median PFS of 9.4 months (ORR, p=0.040; DCR, p=0.037; PFS distribution, p=0.073; OS distribution, p=0.181). In the neuroblastoma subgroup (n=5), the observed ORR was 20.0% and the DCR was 60.0%. A separate historical cohort of 33 heavily pretreated patients receiving bevacizumab, irinotecan, and temozolomide reported an ORR of 9.1% (15); this value is provided solely as clinical context and should not be interpreted as a comparative estimate because of differences in sample size, treatment regimen, prior treatment, and study population. Histology-specific variation is biologically plausible given the genomic heterogeneity documented in Chinese and multi-institutional pediatric solid-tumor cohorts (16, 17), although our subgroup findings remain exploratory. Patients with metastatic or recurrent disease had a lower DCR than those without metastatic or recurrent disease (28.6% vs 80%; p=0.008); PFS distributions also differed between the disease-status groups (p=0.011).

### Treatment regimen considerations

In the anlotinib monotherapy group (Group A, n=4), the observed ORR was 50% and the DCR was 75%; these estimates were based on only four patients. Across the five treatment-regimen groups, no statistically significant differences were detected in ORR (p=0.133), DCR (p=0.454), PFS distributions (p=0.480), or OS distributions (p=0.937). The crude median PFS estimates were not reached, 2.1, 5.6, 2.4, and 3.0 months in Groups A, B, C, D, and E, respectively. The groups were small and markedly unbalanced and differed in tumor type, disease status, prior treatment, and concomitant therapy. Consequently, these unadjusted estimates cannot isolate the effect of any individual anlotinib-containing regimen. For clinical context, Lu et al. (3) reported median PFS estimates of 2.80 months with chemotherapy combinations and 1.13 months with immunotherapy combinations in another small retrospective cohort. These cross-study numerical observations do not establish comparative regimen effectiveness. The regimen-group findings in the present study should therefore be interpreted solely as descriptive and hypothesis-generating.

The DCR was 50% in the group receiving anlotinib plus ICI in our cohort and 66.7% in the corresponding pediatric combination group reported by Lu et al. (3). In adults with advanced non-small cell lung cancer, a phase Ib study of sintilimab plus anlotinib reported an ORR of 72.7% and a DCR of 100% (18). Preclinical neuroblastoma models also showed that anlotinib promoted vessel normalization and a T-cell-inflamed tumor microenvironment and enhanced PD-1 blockade (19). These findings provide a rationale for further study of anlotinib-ICI combinations in pediatric solid tumors.

The DCR was 50% in the targeted therapy combination group (Group E). One patient receiving anlotinib plus bevacizumab maintained SD. This combination has been evaluated prospectively in adults with recurrent high-grade glioma (20), providing clinical precedent for combined targeting of the vascular endothelial growth factor pathway. Larger studies are required to evaluate this strategy in pediatric solid tumors.

### Safety profile and TEAE monitoring

Any-grade and grade 3–4 TEAEs were documented in 97.8% and 84.4% of patients, respectively, indicating a high burden of severe treatment-emergent events. Hematologic TEAEs were the most frequently documented category. Because 86.7% of patients received combination chemotherapy and/or immunotherapy and no formal causality adjudication was performed, these events cannot be attributed specifically to anlotinib. Hematologic abnormalities may also have been influenced by concomitant chemotherapy, previous treatment, the underlying malignancy, or pre-existing abnormalities that could not be systematically reconstructed from the retrospective records.

Regimen-specific TEAE frequencies are presented as numerical observations only. The small and markedly unbalanced treatment groups, differences in concomitant therapies, and absence of formal causality assessment preclude comparative or mechanistic interpretation. Dose reduction or discontinuation of anlotinib was documented in three patients (6.7%); this limited number of dose modifications does not lessen the high observed burden of grade 3–4 TEAEs.

### Dose optimization and practical implications

The LD cohort (n=25) had a numerically higher DCR than the UD cohort (n=18) (48% vs 33.3%) and a numerically longer median PFS (6.2 vs 3.0 months). However, no statistically significant differences were detected across the UD, LD, and HD cohorts in ORR (p=0.692), DCR (p=0.393), PFS distributions (p=0.395), or OS distributions (p=0.787). Dose selection was based on clinical judgment and may have been influenced by age, body weight, tumor type, disease status, prior therapy, concomitant treatment, and anticipated tolerance. The small and unbalanced dose groups therefore permit only unadjusted descriptive comparisons and cannot establish a preferred dose or comparative dose effectiveness. Nevertheless, documenting the dose patterns used in practice together with the corresponding clinical outcome and TEAE data helps define the questions that prospective pediatric dose-finding studies should address. The vascular-normalization framework provides a biological basis for dose- and schedule-dependent effects of antiangiogenic therapy (21), while the optimal biological dose (OBD) concept seeks a dose that provides sufficient biological and antitumor activity with acceptable toxicity (22, 23). The pediatric phase Ia/Ib trial identified a recommended phase II dose of 8 mg for patients weighing <35 kg and 12 mg for those weighing ≥35 kg (8). These findings and prior evidence support prospective weight-stratified dose-finding and exposure-response studies across tumor types and treatment regimens. The present findings do not support routine reduced-dose initiation. Clinical dose selection should remain individualized according to body weight, clinical status, concomitant therapy, and tolerance, with close monitoring and timely dose adjustment.

### Strengths, limitations, and future directions

This study has several strengths. It provides real-world evidence from a Chinese tertiary pediatric oncology center from January 2020 to May 2025, including a high proportion of patients with metastatic or recurrent disease, reflecting the real-world salvage setting at our center. Eligibility required RECIST 1.1-measurable disease, and treatment response and safety were assessed using standardized criteria, including RECIST 1.1 (10), CTCAE v5.0 (12), and MedDRA (13). In addition, outcomes were described across histologic subtypes, disease status, treatment regimens, and actual dose groups, providing clinically relevant reference data for off-label pediatric anlotinib use. By documenting the actual dose categories used in clinical practice alongside clinical outcomes and TEAE frequencies, this study provides descriptive reference data for designing prospective pediatric dose-optimization studies.

This study also has several limitations that should be acknowledged. The retrospective single-center design may introduce information bias and limit generalizability. The exclusion of seven patients with insufficient post-treatment imaging or follow-up information may have introduced selection bias, potentially leading to overestimation of response and survival outcomes and underestimation of the TEAE burden. Selection bias is possible because our tertiary referral setting included a high proportion of patients with metastatic or recurrent disease (77.8%), reflecting a heavy baseline tumor burden. The small sample size and imbalance across treatment groups, especially Groups A, B, and E, limited statistical power; subgroup findings should therefore be interpreted as descriptive observations. The composite disease-status variable may also have obscured clinically relevant differences between metastatic and recurrent disease, while refractory status could not be analyzed separately because it was not consistently available in the retrospective dataset. No multivariable adjustment was performed. Multiple exploratory subgroup comparisons were conducted without adjustment for multiplicity, increasing the possibility of chance findings. Accordingly, the crude subgroup associations may reflect measured or unmeasured confounding related to age at anlotinib initiation, tumor type, disease status, performance status, number of previous treatment lines, anlotinib dose, concomitant therapy, and anticipated tolerance. These analyses cannot isolate the independent effects of anlotinib, dose category, or treatment regimen. Height was not systematically collected, so body surface area-based dose metrics could not be reconstructed reliably. Performance status, uniform tumor stage, separate recurrent and refractory classifications, number of previous treatment lines, detailed previous and concomitant chemotherapy, treatment duration, number of administered cycles, dose intensity, and detailed reasons for reduced-dose selection or treatment discontinuation were not consistently retrievable from the retrospective electronic medical records. RECIST 1.1 may not fully capture CNS-specific response patterns; however, the sensitivity analysis excluding primary CNS tumors yielded a similar qualitative interpretation.

Future research should address these limitations through multicenter prospective studies to characterize long-term clinical outcomes and safety. Specifically, multicenter phase I/II dose-finding studies should compare multiple anlotinib dose levels, including reduced-dose strategies in chemotherapy-containing regimens, with stratification for tumor type and body weight. Exposure-response studies are also needed to link pharmacokinetics with dynamic biomarkers such as vascular endothelial growth factor and to identify tumor- and regimen-specific dose ranges that optimize the balance between antitumor activity and toxicity. These studies should determine whether prospectively assigned lower-dose strategies can reduce the TEAE burden without compromising antitumor activity; the present retrospective findings do not justify routine reduced-dose initiation.

## Conclusion

In this small, heterogeneous, single-center retrospective cohort, pediatric patients receiving anlotinib-containing regimens had an ORR of 8.9%, a DCR of 40.0%, and a median PFS of 5.6 months; OS data remained immature. Grade 3–4 TEAEs were documented in 84.4% of patients and predominantly involved hematologic events. The subgroup findings were exploratory and may reflect selection bias, confounding by indication, tumor heterogeneity, and concomitant therapies; these analyses cannot isolate the independent effect of anlotinib or establish comparative effectiveness among treatment or dose groups. Larger prospective studies are required before comparative treatment or dosing conclusions can be made.

## Data Availability

The datasets analyzed in this study are not publicly available because they contain potentially identifiable clinical information from pediatric patients. Requests to access de-identified data should be directed to the corresponding authors and will be considered subject to institutional and ethical approval.
